# Self-assembly of large-scale gold nanoparticle arrays and their application in SERS

**DOI:** 10.1186/1556-276X-9-114

**Published:** 2014-03-13

**Authors:** Sheng-Qing Zhu, Tong Zhang, Xin-Li Guo, Xiao-Yang Zhang

**Affiliations:** 1 School of Electronic Science and Engineering, Southeast University, Nanjing 210096, People’s Republic of China; 2 Key Laboratory of Micro-Inertial Instrument and Advanced Navigation Technology, Ministry of Education, Nanjing 210096, People’s Republic of China; 3 Suzhou Key Laboratory of Metal Nano-Optoelectronic Technology, Suzhou Research Institute of Southeast University, Suzhou 215123, People’s Republic of China; 4 School of Materials Science and Engineering, Southeast University, Nanjing 211189, People’s Republic of China

**Keywords:** Self-assembly, Gold nanoparticle, SERS, Plasmonics, Localized surface plasmon resonance

## Abstract

Surface-enhanced Raman scattering is an effective analytical method that has been intensively applied in the field of identification of organic molecules from Raman spectra at very low concentrations. The Raman signal enhancement that makes this method attractive is usually ascribed to the noble metal nanoparticle (NMNP) arrays which can extremely amplify the electromagnetic field near NMNP surface when localized surface plasmon resonance (LSPR) mode is excited. In this work, we report a simple, facile, and room-temperature method to fabricate large-scale, uniform gold nanoparticle (GNP) arrays on ITO/glass as SERS substrates using a promoted self-assembly deposition technique. The results show that the deposition density of GNPs on ITO/glass surface increases with prolonging deposition time, and nanochain-like aggregates appear for a relatively longer deposition time. It is also shown that these films with relatively higher deposition density have tremendous potential for wideband absorption in the visible range and exhibit two LSPR peaks in the extinction spectra because the electrons simultaneously oscillate along the nanochain at the transverse and the longitudinal directions. The SERS enhancement activity of these GNP arrays was determined using 10^-6^ M Rhodamine 6G as the Raman probe molecules. A SERS enhancement factor as large as approximately 6.76 × 10^6^ can be obtained at 1,363 cm^-1^ Raman shift for the highest deposition density film due to the strong plasmon coupling effect between neighboring particles.

## Background

Surface-enhanced Raman scattering (SERS) has been considered as a highly sensitive and convenient analytical tool to detect chemical and biological molecules [1-7]. SERS provides an extreme signal enhancement over traditional Raman spectrum intensity due to the effect of localized surface plasmon resonances (LSPR), which is an optical phenomenon arising from the collective oscillation of conduction electrons in a noble metallic nanostructure when the electrons are disturbed from their equilibrium positions [8,9]. The plasmonic behaviors of the structures (e.g., the position of resonant peaks, transmission pass-bands, and the magnitude of the optical-field enhancement) are highly sensitive to their size, shape, composition, and surrounding medium [10,11]. Moreover, the distance of sub-10 nm between neighboring noble metal particles is also an important factor that affects the amplification ability of SERS signal because the plasmonic electromagnetic field obtained from interparticle plasmon coupling, known as ‘hot spots’ or ‘hot junctions', is significantly larger than that obtained from isolated particles [12,13].

To obtain tremendous SERS signal enhancement ability, numerous available approaches such as electron beam lithography (EBL) [14,15], nanoimprintation [16,17], nanosphere lithography (NSL) [18], mask-assisted deposition (MAD) [19], vacuum evaporation, and other strategies have been proposed to fabricate well-ordered or random nanostructures [20,21], which composed of uniform noble metal (Au or Ag) nanoparticles. EBL method can completely control the formation, shape and size of the nanostructures for the design of metallic films with unique LSPR spectra but is too expensive for practical applications. Nanoimprintation, NSL, and MAD methods can provide large-scale uniform noble metallic structure array, but the preparation process is complicated and the gap between particles cannot be reduced to sub-10 nm. Vacuum evaporation should be a simple, low-cost, and large-scale approach to produce nanoisland array but it cannot control the shape of the nanostructures. Currently, the self-assembly method is widely used to fabricate highly large-scale-ordered two-dimensional noble metal particle films (Au or Ag) consisting of metal nanoparticles such as nanosphere, nanorod, nanocube, and nanotriangular on ITO/glass or Si substrates [22-26]. However, such self-assembly method usually require complicated preparation processes and special substrate surface modifications. Therefore, exploring a new simple method that directly assembles large-scale NMPs on a no-special surface-treated substrate is still a formidable challenge.

In this paper, we proposed a promoted self-assembly method for fabricating gold nanoparticle (GNP) arrays onto ITO/glass substrate surface. This method has advantages of being simple, room-temperature preparation, no special modification of substrate surface, and having the capability to tune the GNP deposition density through prolonging deposition process time. Furthermore, we find that after a deposition time longer than 6 days, gold nanochains appear. This kind of nanochains has two strong LSPR peaks because electrons simultaneously oscillate along the transverse and the longitudinal directions. Due to the stronger LSPR effect of the films, we use Rhodamine 6G as probe molecules to estimate the enhancement capability of the films to SERS signal. Finally, we demonstrate the excellent Raman signal enhancement on these metallic films and found that a SERS enhancement factor as large as approximately 6.76 × 10^6^ can be obtained. Consequently, our experiment indicates that this facile self-assembly method may be a promising strategy to prepare large-scale, inexpensive, highly sensitive SERS substrates.

## Methods

### Chemicals and reagents

Gold chloride trihydrate (HAuCl_4_ · 3H_2_O, >99.9%), sodium borohydride (NaBH_4_, >96%), cetyltrimethylammonium bromide (CTAB, >99.0%), polyvinyl-pyrrolidone (PVP, >99.0%), and ascorbic acid (AA, >99.5%) were purchased from Sinopharm Chemical Reagent Co., Ltd. (Shanghai, China) and used without further purification. Ultrapure water (resistivity >18.0 MΩ cm) was used throughout the experiments.

### Preparation of GNP solution

The GNP solution was prepared using a modified seed-mediated approach according to Murphy's method [27,28]. The synthesis process of GNP solution contains two steps: seed synthesis and particle growth process. In a typical procedure, the gold seed solution was prepared by the addition of a freshly prepared ice-cold 0.3 mL of aqueous 0.01 M NaBH_4_ solution into an aqueous mixture solution which composed of 0.125 mL of an aqueous 0.01 M HAuCl_4_ solution and 3.75 mL of an aqueous 0.1 M CTAB, followed by rapid inversion mixing for 2 min. The resulting seed solution was kept at room temperature (approximately 25°C) for 1 h before use. The growth solution was prepared by the sequential addition of 8 mL of 0.1 M CTAB, 1 mL of 0.01 M HAuCl_4_, and 3 mL of 0.1 M AA into 38 mL of deionized water. Amount of 4.175 mL of the CTAB-stabilized seed solution was diluted to 10 mL by adding deionized water, and then 10 μL of the diluted solution was added into the growth solution. The resulting solution was gently stirred using a magneton for 10 s and then left undisturbed overnight. After finishing the growth process of the GNP solution, the solution was centrifuged at 14,000 rpm for 10 min and then redispersed into the deionized water to reduce the concentration of redundant reactants in the solution.

### Self-assembly of large-scale GNP arrays on ITO/glass substrate

Large-scale GNP arrays on ITO/glass substrates were prepared according to the modified method we have previously reported [29]. In a typical process, 2 mL of 0.01 M PVP and 1.5 mL of 0.1 M AA were added into the GNP solution and stirred strongly subsequently. Four pieces of ITO/glass substrates, treated by detergent, acetone, and deionized water in sequence, were immersed into the modified GNP solution for 2, 4, 6, and 8 days respectively. Thus, large-scale GNP arrays were consciously assembled onto the surface of the ITO/glass substrates with the different deposition density. Compared with the previous works, the main feature of this self-assembly method is to be able to directly deposit gold nanoparticles on ITO/glass substrates without special substrate surface modifications, while in previous work, GNPs usually assembled on the substrate surface functionalized with aminopropyltriethoxylsilane [23,25], hydrofluoric acid, or C_18_ alkyl chains [26]. In addition, another advantage of this method is that the density of GNPs deposited on ITO/glass can be conveniently tuned by deposition time. The four substrates with self-assembled GNP arrays (deposited for 2, 4, 6, and 8 days) are denoted as samples A, B, C, and D.

### Preparation of SERS substrates

Rhodamine 6G (R6G) was used as the probe molecular for Raman detection. Amount of 10 μL of 10^-6^ M R6G (in ethanol) was dropped onto the surface of the four samples with different GNP arrays, respectively, and blow dried for SERS measurement.

### Characterization of materials

The morphological features of the GNPs in solution and GNP arrays on ITO/glass substrates were characterized by Tecnai G2 transmission electron microscope (TEM) and Quanta 400 FEG field emission scanning electron microscope (SEM) (FEI Company, Hillsboro, OR, USA). Samples for TEM were prepared by placing a drop (approximately 5 μL) of GNP solution onto a carbon-coated copper grid and dried at room temperature. Extinction spectra were collected on a UV–vis-near-infrared spectrophotometer (UV2100). Raman spectra were obtained using a confocal microprobe Raman system (HR 800) equipped with a holographic notch filter and a CCD detector. A long working distance × 50 objective was used to collect the Raman scattering signal. The laser beam size focused on the samples is 1.5 μm in diameter. An Ar laser (514 nm) and a He-Ni laser (633 nm) were used for the excitations.

## Results and discussion

### Synthesis and characterization of the GNPs

**Figure 1:**
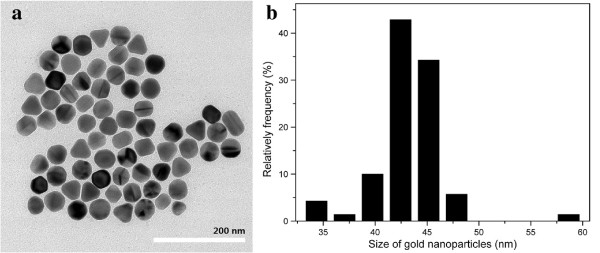
TEM image of GNPs (a) and particle number distribution for related TEM image (b).

**Figure 2:**
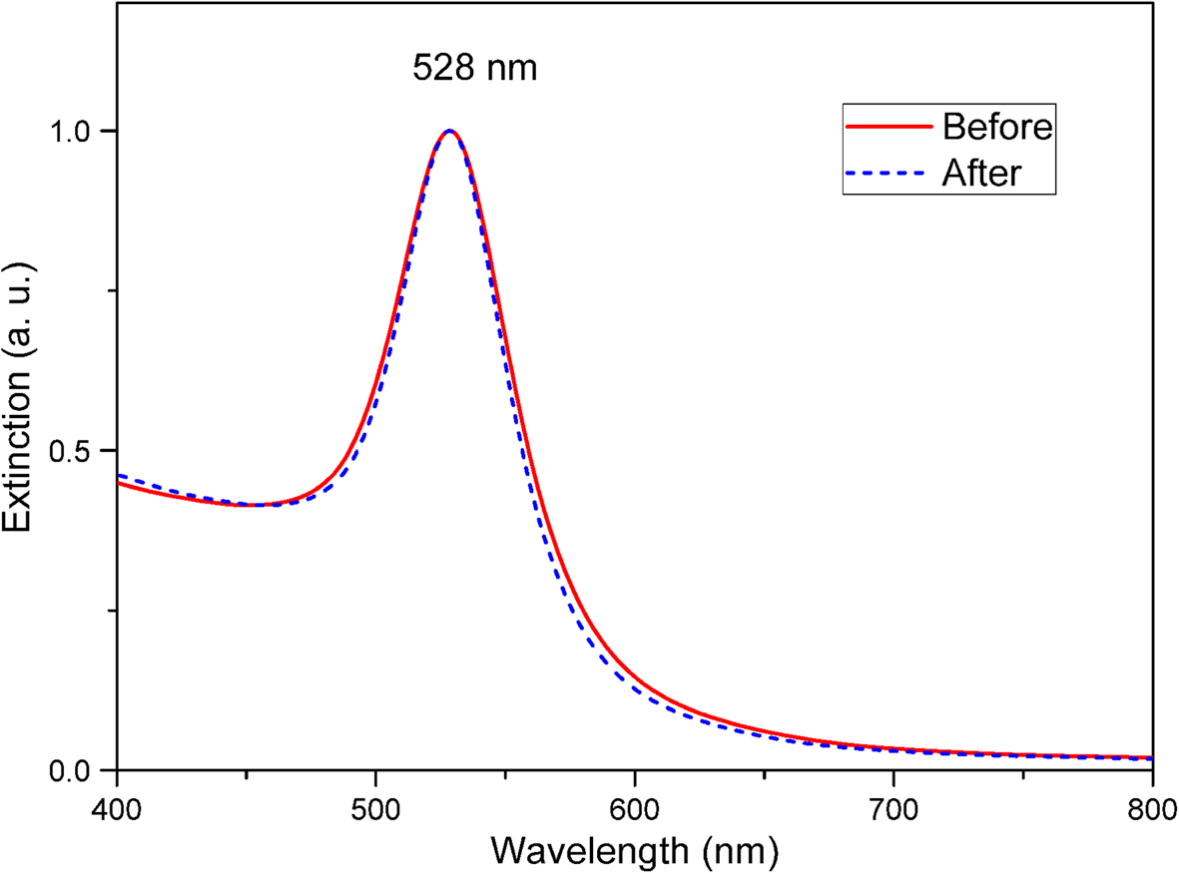
Normalized extinction spectra of the GNP solution before and after adding PVP and AA.

In our approach, the GNP solution was used as the raw material source for GNP arrays deposition. We modified the concentration of the reactants as Murphy has reported [27], and CTAB-stabilized GNPs were obtained in deionized water. Figure 1 shows the TEM image of GNPs in water solution before adding PVP and ascorbic acid. It is found that the GNPs are single crystals with a nearly quasi-spherical shape and well dispersed in the solution due to the positive surface charges caused by the stabilizing agent CTAB [30]. From the particle size distribution chart (Figure 1b), the average size of GNPs is about 43 nm, which is dependent on the amount of gold seeds added during the synthesis process. Then, we also examined the plasmon resonance characteristic of GNP solution and found that the solution has a single narrow LSPR peak at 528 nm (Figure 2 red solid line), and this result is consistent with the previous works [31]. The electron transition between the 5d^10^ level and unoccupied conduction bands leads to the LSPR extinction spectrum for monodispersed GNPs in solution [32].

### Micro-morphological and optical characterization of the self-assembled GNP arrays

**Figure 3:**
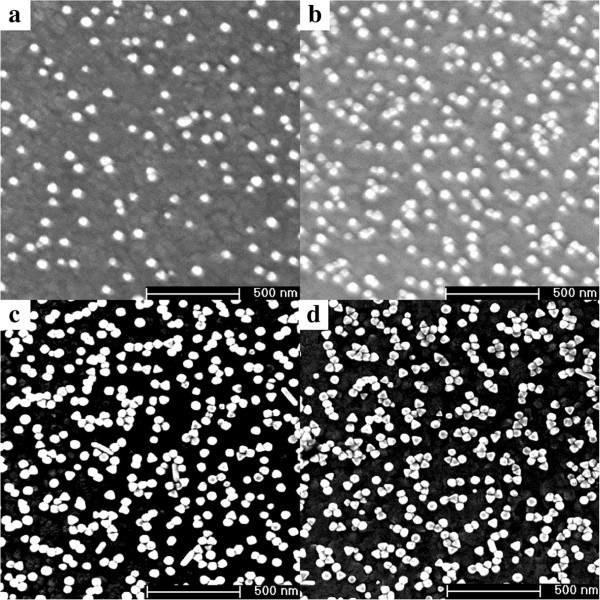
**SEM images of GNP arrays with different deposition times. (a)** 2 days. **(b)** 4 days. **(c)** 6 days. **(d)** 8 days.

In the self-assembly process, we utilize a strategy using the chemical ingredients, including PVP and AA, to assemble the GNP arrays onto the surface of ITO/glass substrates. The ITO/glass substrates are used for the convenience of SEM detection because of their electro-conductivity. AA has been found to be a key ingredient for the self-assembly production of large-scale GNP arrays in aqueous solution because it can modify the surface activity of GNPs significantly. PVP is usually used as a shape-directing agent or stabilizer during the deposition procedures of GNPs [29]. Figure 3 shows SEM images of the four samples A to D in which GNP arrays assembled on ITO/glass substrates with different deposition time of 2, 4, 6, and 8 days, respectively. The most obvious change is that the density of GNPs assembled on the ITO/glass substrates increased correspondingly with the increase of the deposition time. Beside this, the micro-morphology of the GNP arrays also varies with the different deposition time. From the Figure 3c,d, some special nanostructures of nanochains emerge on the ITO surfaces of samples C and D in the case of relatively longer deposition time compared with the samples A and B. The nanochain structures usually exhibit some novel optical properties, such as LSPR peak shifting and more enhanced electromagnetic field [33,34]. Moreover, to determine whether the nanochain structures are produced in the GNP solution or only on the surface of ITO/glass substrates after adding PVP and AA, we examined the extinction spectra of GNP solution again after adding the two chemical ingredients (Figure 2 blue dashed line). It is found that the spectrum shows the similar optical property and only differs in full width at half maximum because of a minor change of refractive index of the environment. This indicates that the nanochain structures are only produced on ITO/glass surface rather than in the GNP solution.

**Figure 4:**
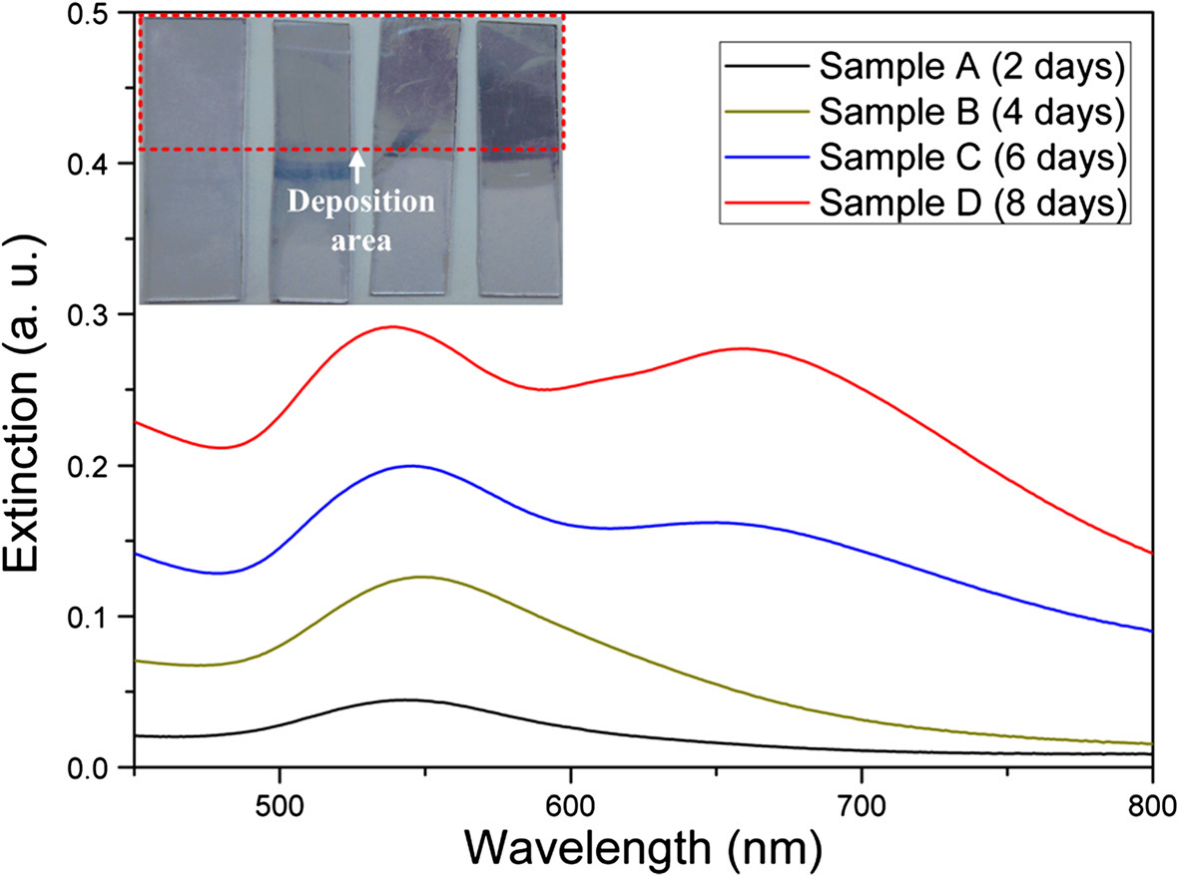
**Extinction spectra of the samples A to D, respectively.** The inset shows the photograph of the four samples.

To study the effect of the nanochains on the optical property of GNP arrays, we resorted to using a UV–vis-near infrared spectrophotometer to achieve the extinction spectra of samples A to D. Figure 4 exhibits the extinction spectra of the four samples A to D, respectively. The inset shows the photograph of the samples A to D from left to right. The colors of the samples gradually vary from pale red to purple as the GNPs deposition density increases. The GNP arrays withrelatively shorter deposition time (samples A and B) exhibited relatively weaker LSPR and single peak at about 546 nm, which is different from the peak position at 528 nm in solution because the surrounding environment refractive index changes [29]. In contrast, when the deposition time is prolonged to 6 or 8 days (samples C and D), the GNP arrays exhibited two absorbance bands. One was near 546 nm and the other absorbance band red-shifted to 659 nm. The multiple extinction peaks had led to wideband absorption in the visible range [35] and caused the color of the films to get deeper compared with samples A and B. As evident in the SEM images of samples C and D, no perfect nanorods or nanowires were obtained on ITO/glass surfaces. Therefore, the presence of LSPR peak at long wavelength could be directly related to the nanochain structures on the ITO/glass surfaces of samples C and D. This result is consistent with the Umar's work [33], and the LSPR peak at 659 nm is attributed to the longitudinal oscillation of the free electron system along the axis of the nanochain structures, which is related to the plasmonic coupling between the neighboring nanoparticles [33,36]. However, there were actually not only nanochains but individual GNPs also existing on samples C and D. Therefore, the LSPR peak at the short wavelength can be attributed to two contributions: one is the LSPR effect of the individual GNPs and the other is caused by the transverse surface plasmon excited by the free electron cloud which is oscillating perpendicular to the axis of the nanochain structures. This is the reason that the peak at long wavelength (approximately 659 nm) is not as intense as the one at short wavelength (approximately 546 nm).

### SERS analysis of the self-assembled GNP arrays

**Figure 5:**
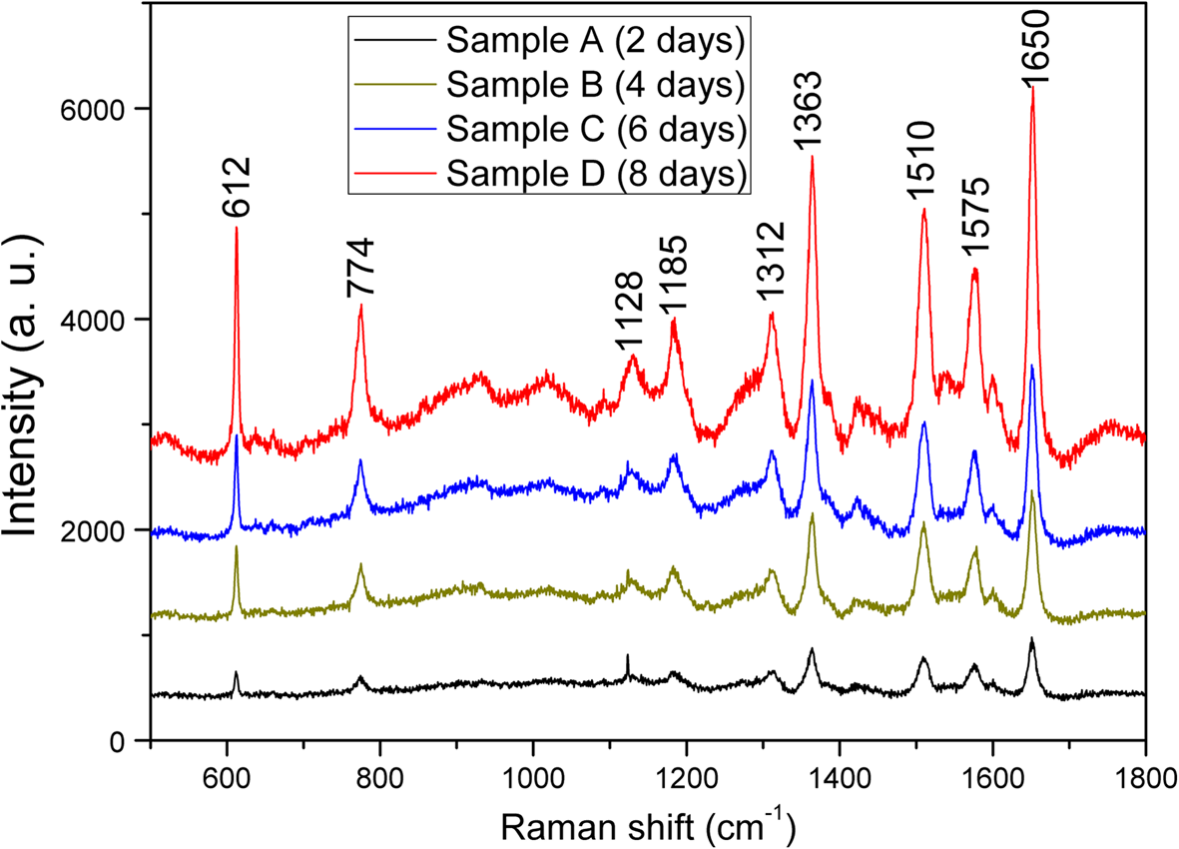
**SERS spectral comparison of 10**^**-6**^ **M R6G adsorbed on GNP arrays under 514 nm excitation.**

It is well known that films composed of gold nanostructures exhibit a strong SERS effect [37,38]. Raman spectroscopy is not only a powerful analytical technique in composition analysis but also an effective testing tool to examine the LSPR effect of GNP thin films. The Raman spectra have been intensively used in the field of identification of organic molecules from their vibration spectra at very low concentrations [39,40]. We investigated the SERS activity of the four obtained GNP arrays on ITO/glass substrates, and an ethanol solution (10^-6^ M) of R6G was used as the Raman probe molecules because they have been extensively studied in previous literatures [41]. R6G is a strongly fluorescent xanthene derivative that is a yellowish heterocyclic compound and shows a molecular resonance Raman effect when excited into its visible absorption band. In this study, we use an Ar laser (514 nm) and a He-Ni laser (633 nm) as the excitation source because their emission wavelengths are close to the surface plasmon resonance arising from the surfaces of the four samples (depicted in Figure 4). Figure 5 shows the SERS spectra of the GNP arrayscapped by R6G molecules on samples A to D at an excitation wavelength of 514 nm. Salient surface-enhanced characteristic peaks of R6G molecules on the samples can be seen noticeably. The peak at 612 cm^-1^ is due to plane bending of the C-C-C ring, whereas the band at 774 cm^-1^ has been assigned to out-of-plane bending of the hydrogen atoms of the xanthene skeleton [42]. The peak at 1,185 cm^-1^ is associated with C-C stretching vibrations, and those at 1,312, 1,363, 1,510, 1,575, and 1,650 cm^-1^ correspond to aromatic stretching vibrations agreeing with those reported in the literatures [1,43]. Comparing these Raman signals with the extinction spectra depicted in Figure 4, it can be observed that the intensity of Raman signals is related to LSPR effect of the GNP arrays and increase with the increment of resonance peak at 546 nm.

To investigate the Raman enhancement effect of R6G on these GNP arrays quantitatively, the enhancement factor (EF) values were calculated using the methods reported by Van Duyne [44]. The EF is defined as EF = (*I*_SERS_/*C*_SERS_)/(*I*_0_/*C*_0_), where *I*_0_ and *C*_0_ are the peak intensity of the Raman signal and concentration for the regular Raman measurement with 0.1 M R6G solution on ITO/glass substrate, respectively; *I*_SERS_ and *C*_SERS_ are the peak intensity and concentration, i.e., 1 × 10^-6^ M used in this experiment, for the SERS measurement, respectively. Here, we chose the Raman band at 1,363 cm^-1^ and calculated the enhancement factor for each sample. The results are listed in Table 1. This calculation is based on the fact that the intensity of SERS is proportional to the number (or concentration) of molecules under otherwise equal conditions. The EF obtained from the sample D can reach to 5.83 × 10^6^ which is larger than that of the samples A to C. However, we suggest that the major reason for determining the values of EFs did not relate to the LSPR coupling effect in nanochain structures, because the extinction peak at 546 nm around the laser exciting wavelength (514 nm) is ascribed to the plasmon resonance of individual particles and transverse surface plasmon mode of nanochain structures, while only the absorbance band at 659 nm is the result of LSPR coupling between neighboring particles in nanochains.

**Table 1:** Enhancement factor of R6G in each structure under 514 nm excitation

	**Sample A**	**Sample B**	**Sample C**	**Sample D**
EF	9.32 × 10^5^	2.05 × 10^6^	3.44 × 10^6^	5.83 × 10^6^

**Figure 6:**
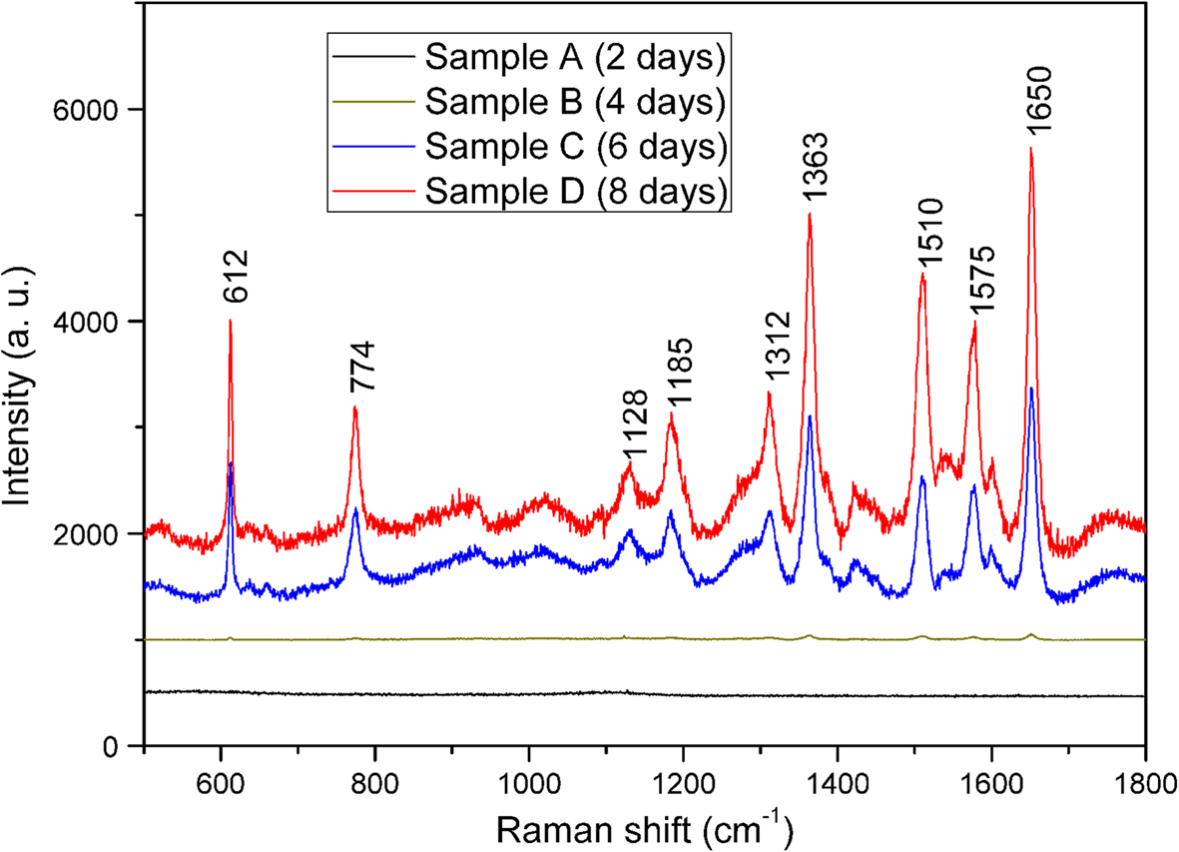
**SERS spectral comparison of 10**^**-6**^ **M R6G adsorbed on GNP arrays under 633 nm excitation.**

**Figure 7:**
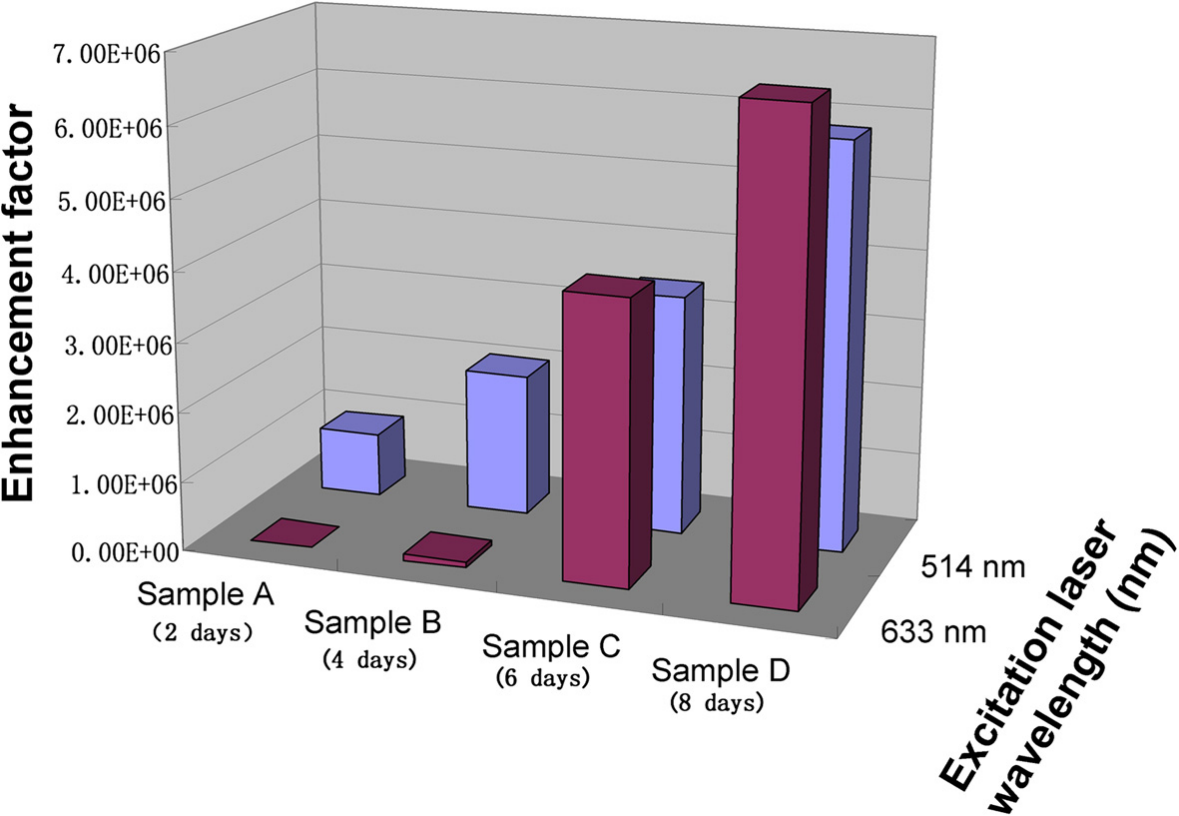
**Empirical SERS enhancement factors obtained on 1,363 cm**^**-1 **^**Raman mode under different laser excitations.**

To further understand this LSPR coupling effect on Raman enhancement that occurs on the nanochain structures which only exist on the samples C and D, we measured the SERS signals again with 10^-6^ M R6G solution using another excitation source (633 nm). These Raman measurements were performed under the same conditions except the excitation wavelength. From the Raman enhancement, one can see that the EFs obtained from samples C and D are much higher than that obtained from samples A and B (Figure 6). The quantitative enhancement factors obtained from all samples are shown in Table 2. The highest EF can reach to 6.76 × 10^6^ for sample D and an EF value of 4.04 × 10^6^ also can be achieved for sample C. The result also indicates that this kind of films composed of nanochain structures has great signal amplification ability in SERS measurement at multiple excitation laser wavelengths of 514 and 633 nm, while previously reported SERS substrate usually amplify Raman signal at only one excitation wavelength. However, for the samples A and B, they show much weaker abilities of SERS signal amplification and even show no discernible Raman peak of R6G for sample A. In order to compare the relationship between the SERS amplification and the LSPR effect at different excitation wavelengths, we exhibited the two sets of the SERS results on a column chart at the excitation wavelengths of 514 and 633 nm (Figure 7). Obviously, the EF is a function of excitation wavelength and is dependent on the density of GNPs-deposited ITO/glass surface. However, a very interesting one is that the intensity of the extinction peaks around 633 nm is weaker than that around 514 nm (Figure 4), but EFs under 633 nm excitation are much higher than that under 514 nm excitation for samples C and D. This phenomenon indicates that the excessive Raman enhancement is associated with the nanochain structures on samples C and D, because the electromagnetic field in the sub-10-nm gap regions between neighboring particles in nanochains can be extremely increased by several orders of magnitude, producing hot spots that are not present in isolated spherical nanoparticles [12,45,46]. This is the reason that the optical behaviors of samples A and B are not similar to samples C and D, and the Raman signal enhancement under 633 nm excitation is much lower than that under 514 nm excitation.

**Table 2:** Enhancement factor of R6G in each structure under 633 nm excitation

	**Sample A**	**Sample B**	**Sample C**	**Sample D**
EF	-	8.92 × 10^4^	4.04 × 10^6^	6.76 × 10^6^

## Conclusion

In summary, we report a simple method to assemble gold nanoparticle arrays on ITO/glass substrates using two chemical ingredients of PVP and ascorbic acid. The micro-morphology varies with the increase of deposition time, and nanochain structures appear when prolonging to 6 or 8 days. These nanochain structures exhibit high Raman signals of R6G due to the strong LSPR effect in the sub-10-nm gap regions. This design of GNP arrays with a highly sensitive SERS-active property may provide a new framework for the fabrication of large-scale SERS-based sensors.

## Competing interests

The authors declare that they have no competing interests.

## Authors’ contributions

S-QZ, TZ, X-LG, and X-YZ carried out the design and the characterization of GNP thin film. S-QZ performed the SERS analysis and drafted the manuscript. TZ, X-LG, and X-YZ read and contributed to the improvement of the manuscript. All authors read and approved the final manuscript.
